# *Klebsiella pneumoniae* bacteraemia epidemiology: resistance profiles and clinical outcome of King Fahad Medical City isolates, Riyadh, Saudi Arabia

**DOI:** 10.1186/s12879-023-08563-8

**Published:** 2023-09-05

**Authors:** Taghreed A. Hafiz, Sarah Alanazi, Shahad S. Alghamdi, Murad A. Mubaraki, Waleed Aljabr, Nouf Madkhali, Sarah R. Alharbi, Khalifa Binkhamis, Fawzia Alotaibi

**Affiliations:** 1 Clinical Laboratory Sciences Department, College of Applied Medical Sciences, King Saud University, Riyadh, 12372 Saudi Arabia; 2 Microbiology Department, Regional Laboratory and Central Blood Bank, Al-Baha, 65715 Saudi Arabia; 3 Research Center, King Fahad Medical City, Riyadh, 11525 Saudi Arabia; 4 Molecular Department, King Fahad Military Medical City, Dhahran, 31932 Saudi Arabia; 5 Pathology Department, College of Medicine, King Saud University, Riyadh, 12372 Saudi Arabia

**Keywords:** *Klebsiella pneumoniae*, Bacteraemia, Nosocomial infection, Multi-drug resistant, Pan-drug resistant

## Abstract

**Background and objectives:**

*Klebsiella pneumoniae* (*K. pneumoniae*) is the second leading cause of community-acquired and hospital-acquired gram-negative bloodstream infection (BSI). This study aimed to assess the epidemiological and microbial-resistance characteristics and clinical factors associated with *K. pneumoniae* BSI in Saudi Arabia.

**Materials and Methods:**

Data of 152 *K. pneumoniae* isolates diagnosed between January 2019 and January 2020 at King Fahad Medical City, Riyadh, Saudi Arabia were evaluated retrospectively. Clinical records of the patients were collected and analysed statistically.

**Results:**

In total, 152 cases of *K. pneumoniae* BSI were identified. Adult patients (66.4%) were at a higher risk of developing the infection than paediatric patients (33.6%). The rate of infection was slightly higher in women than in men. Neurological disorders were the predominant underlying conditions for the acquisition of *K. pneumoniae* BSI, at all ages. Most of the deceased patients were adults with multi-organ dysfunction. *Klebsiella pneumoniae* showed disturbing resistance to amoxicillin-clavulanate and cefuroxime (72.4%), ceftazidime (67.8), cephalothin (76.3%), and to Carbapenems (36.1%).

**Conclusions:**

The impact of *K. pneumoniae* BSI was seen not only at the patient level, but also at the community level, and was related to multi-drug resistant infection. These findings provide a better understanding of microbial resistance and its association with patient clinical outcomes.

## Introduction

*Klebsiella pneumoniae*, a normal commensal organism living on human mucosal surfaces, including the gastrointestinal tract and oropharynx, can invade other tissues and cause severe infections [1]. It is the second leading cause of bloodstream infections (BSI) caused by gram-negative bacteria, after *Escherichia coli* [2]. BSI can occur as a primary infection with no identifiable source. However, it is typically caused by spread from a known source into the bloodstream. The urinary tract, gastrointestinal tract, intravenous or urinary catheters, and respiratory sites are common sources of secondary BSI [3].

Several investigations have indicated that diabetes, hepatobiliary illness, and neoplastic disease were associated as risk factors to around 50% of community-acquired *K. pneumoniae* [4, 5] Lin Y et al. (2010), on the other hand, discovered that chronic lung illness was the primary underlying disease, which may be supported by the older age of their research cohort. Prior antibiotic usage, as well as the use of invasive procedures such as an endotracheal catheter, bladder catheter, and intravenous catheter, are key risk factors for developing such infection [6].

Over the past two decades, *K. pneumoniae* has become clinically significant owing to its increased antibiotic resistance, propensity to develop antibiotic resistance, and ability to produce serious outcomes [7, 8]. More recently, it has been identified as a major source of hospital-acquired pneumonia, accounting for approximately. 10% of all hospital-acquired infections, second among gram-negative bacteria [9]. Multidrug-resistant (MDR) *K. pneumoniae* is one of the main factors responsible for nosocomial infections, including pneumonia, urinary tract infections, and bloodstream infections (BSIs); its production of extended-spectrum β-lactamases (ESBL) and carbapenemases causes high mortality rates (40–50%), primarily among critically ill and immunocompromised patients [10]. The emergence of extensively drug-resistant (XDR) and pan-drug-resistant (PDR) strains, via continuous accumulation of antibiotic-resistant genes, protects *K. pneumoniae* from all available antimicrobials [11, 12].

The increasing prevalence of multidrug-resistant *K. pneumoniae* is a global health concern listed by the World Health Organization (WHO) as a critical priority. Unfortunately, in Saudi Arabia, research findings regarding *K. pneumoniae* infections are consistent with the global emergence of drug-resistant strains [13, 14]. There have been multiple reports of NDM and OXA-48 carbapenemases producing *K. pneumoniae* in Saudi Arabia [15, 16], and more recently the first case of KPC producing *K. pneumoniae* has been reported [17]. Furthermore, a study of ICU patients revealed a high prevalence of drug-resistant Enterobacteriaceae infections, which are related to a high mortality rate. Out of the 227 Enterobacteriaceae cultures included in that study, 60% were either MDR (n = 130) or XDR (n = 8) infections, with no PDR cultures; *K. pneumoniae* represented (33%) of the MDR/XDR cultures [18].

Such reports emphasise the necessity of continuous monitoring of the antimicrobial resistance of bacterial isolates, as this can be used to provide guidance to clinicians in the application of empirical treatment. This retrospective study investigated *K. pneumoniae* bacteraemia cases at the King Fahad Medical City (KFMC) in Riyadh, Saudi Arabia. The primary objectives were to evaluate the epidemiological patterns, determine the antimicrobial resistance profiles of the isolates, and clarify their association with patients’ clinical outcomes.

## Materials and methods

### Study design and setting

This retrospective study was conducted over one year (January 2019 to January 2020) at KFMC, which has a capacity of 1200 beds. A total of 152 *Klebsiella pneumoniae* isolates from blood clinical samples were analysed. The clinical history of 152 patients was included in this study.

### Data collection

In total, 152 samples of *Klebsiella pneumoniae* were collected from blood (central and peripheral line blood). The following were the included collection categories: (A) age (paediatric or adult). (B) Ward or clinic to which the patient was admitted (emergency, ICU, ward, or outpatient clinic). (C) blood sample source and location or site; and (D) bacterial-resistance category (susceptible, Extended-spectrum β-lactamases (ESBL), and Carbapenem-Resistant strains). Any growth other than that of *K. pneumoniae* was excluded from the study. Clinical history was collected from the KFMC database for paediatric and adult patients admitted to ICU. The clinical history collected for ICU patients included different criteria: (1) if present, the type of co-infection; (2) exposure to carbapenem or other antibiotics in the past 14–30 days; (3) renal dialysis at isolation or not; (4) mechanical ventilation or not; (5) chronic diseases such as diabetes mellitus (DM), hypertension, renal disease, or malignancy; (6) presence of clinical symptoms such as fever, gastrointestinal tract (GIT) symptoms, or respiratory symptoms; (7) presence of wound or urinary tract infection; (8) presence of bacteraemia or septicaemia; and (9) clinical outcomes and additional notes, if available.

### *Klebsiella pneumoniae* identification and antimicrobial susceptibility testing

All isolates were presumptively identified as *Klebsiella* species, using a Phoenix BD instrument (Becton Dickinson Diagnostic Systems, Sparks, MD, USA) for full identification and sensitivity testing. We included only patients whose isolates were definitively identified as *K. pneumoniae*. Antimicrobial sensitivity testing (AST) was performed for the following antibiotics: ampicillin (AMP), amoxicillin-clavulanate (AMC), piperacillin-tazobactam (TZP), imipenem (IPM), meropenem (MER), ertapenem (ETP), cephalothin (CEF), cefuroxime (CXM), ceftazidime (CTZ), cefoxitin (FOX), cefepime (CFPM), cefotaxime (CTX), ceftriaxone (CRO), ciprofloxacin (CIP), levofloxacin (LVX), gentamicin (GM), amikacin (AMK), tigecycline (TGC), colistin (COL), and trimethoprim-sulfamethoxazole (TMP-SMX). Susceptibility was classified as follows: susceptible, intermediate, or resistant. Confirmation of resistant isolates was performed using Microbroth dilution. Additional tests of disc diffusion or gradient diffusion (Etest) methods were performed using Mueller-Hinton agar which were then incubated in ambient air at 35 ℃ for 16–20 h. For interpretation, CLSI M100 Interpretive Document for Enterobacterales. EUCAST was used for the interpretation of tigecycline activity.

### Statistical analysis

The data were analysed using GraphPad Prism version 9.3.1. Descriptive analysis, using contingency tables and graphs, was used to illustrate the following data: age divisions, sex, ward/clinic, sample source, and sample site. The descriptive data are expressed as absolute numbers (*n)* and percentages. *P* < 0.05 was considered statistically significant. Relative risk (RR) was computed to demonstrate how much the risk variables raised the risk of mortality following a study by Hafiz et al. (2022) [19].

### Ethical approval

This project was approved by the institutional review board (IRB) of KFMC. Consent was obtained from KFMC according to the ICH GCP ethical code (IRB approval number 20-164E). Informed consent was obtained from all the participants and from the legal guardians of the participants who were below 16 years of age.

## Results

### Demographic and clinical characteristics of patients infected with *K. pneumoniae* BSI

During the study period, 152 incident BSI cases were identified as caused by *K. pneumoniae* isolated from the central or peripheral venous catheter. Approximately two-thirds (66%) of the study population were aged > 15 years. Females were fairly probable as males to be infected with *K. pneumoniae* BSI. Among the incident *K. pneumoniae* bloodstream infections, 53 isolates were classified as ESBL strains, 55 as Carbapenem-Resistant strains, and 44 as susceptible strains. More than half of the *K. pneumoniae* isolates originated from critical care wards, such as ER and ICU wards (Table 1).

**Table 1:** Demographic and clinical characteristics of patients with *Klebsiella pneumoniae* bloodstream infection

Characteristic	Patients (*n* = 152)
*Gender*, n (%)	
Male	73 (48)
Female	79 (52)
*Age group*, n (%)	
Paediatric patient	51 (33.6)
Adult patients	101 (66.4)
*Blood specimen source*, n (%)	
Peripheral line	77 (50.7)
Central venous line	75 (49.3)
*Category of multi-drug resistance, n (%)*	
ESBL^1^ strains	53 (34.87)
Carbapenem-Resistant strains	55 (36.18)
Susceptible strains, n (%)	44 (28.95)
*Type of ward/ clinic*, n (%)	
ICU^2^	63 (41.5)
ER^3^	23 (15.1)
Other	66 (43.4)

Data are presented as number of patients (*n*), with the corresponding percentage in parentheses (%). ^1^ESBL, Extended-spectrum β-lactamases; ^2^ICU, Intensive care unit; ^3^ER, Emergency ward

### Clinical manifestations among adult and paediatric patients infected with *K. pneumoniae* BSI

Univariate analysis was conducted to compare clinical manifestations between paediatric and adult patients with *K. pneumoniae* bloodstream infections (Table 2). Paediatric patients were substantially more likely to develop septicaemia than adults (*P* < 0.0001, 56.9% vs. 21.8%, respectively). However, septic shock was significantly more frequent in adult patients (*P* = 0.0092). In adult patients, *K. pneumoniae* BSI and comorbidities, such as diabetes mellitus, hypertension, malignancy, chronic kidney disease, and ischaemic heart disease, were significantly associated (*P* < 0.0001, *P* < 0.0001, *P* = 0.0078, *P* = 0.0021, and *P* = 0.0004, respectively). Notably, paediatric patients with acute respiratory distress syndrome were slightly more vulnerable to *K. pneumoniae* BSI than adult patients (*P* = 0.040).

**Table 2:** Clinical manifestations among adult and paediatric patients infected with *Klebsiella pneumoniae* bloodstream infection

	Age group				
Characteristic	Children (n = 51)		Adults (n = 101)		*P* value
*Clinical presentation/complications*, n (%)					
Fever	27 (52.94%)	48 (47.52%)			0.6071
Septicaemia	29 (56.86%)	22 (21.78%)			< 0.0001****
Septic shock	4 (7.84%)	26 (25.74%)			0.0092**
Multi-organ dysfunction	3 (5.88%)	6 (5.94%)			> 0.9999
Cardiogenic shock	0 (0.00%)	5 (4.95%)			0.1688
Wound infection	3 (5.88%)	6 (5.94%)			> 0.9999
Urinary tract infection	7 (13.73%)	19 (18.81%)			0.5001
Gastrointestinal infection	8 (15.69%)	28 (27.72%)			0.1103
Respiratory infection	22 (43.14%)	33 (32.76%)			0.2160
*Underlying disease*, n (%)					
Diabetes mellitus	4 (1.96%)	58 (57.43%)			< 0.0001****
Hypertension	5 (9.80%)	52 (51.49%)			< 0.0001****
Malignancy	11 (21.57%)	44 (43.56%)			0.0078**
Renal disease	6 (11.76%)	14 (13.86%)			0.8040
CKD^1^	1 (1.96%	20 (19.80%)			0.0021**
ARDS^2^	9 (17.65%)	6 (5.94%)			0.0400*
IHD^3^	0 (0.00%)	19 (18.81%)			0.0004***
Neurological disorders	41 (80.39%)	88 (87.13%)			0.3384
Hypothyroidism	0 (0.00%)	4 (3.96%)			0.3011

Data are presented as number of patients (*n*) with the corresponding percentage in parentheses (%). * P < 0.05; The statistical significance was indicated by a (*) symbol and the number of * represents the strength of the significance difference. ^1^CKD, Chronic kidney disease; ^2^ ARDS, Acute respiratory distress syndrome; ^3^IHD, Ischemic heart disease

### Clinical outcome of patients infected with *K. pneumoniae* BSI

We conducted a univariate analysis to compare the outcome of all patients with *K. pneumoniae* bloodstream infection. Table 3 shows the relative risks (RRs) of mortality and 95% confidence intervals, demonstrating the strength of the associations between the risk factors and mortality. To ensure an accurate comparison between patients, we excluded four patients because they were transferred to another medical facility. Of the total patients with *K. pneumoniae* BSI (n = 148), the overall mortality rate was 32.4% (48/148 patients). Univariate analysis revealed many risk factors associated with mortality (ranked from highest to lowest significance): mechanical ventilation, multi-organ dysfunction, septic shock, gastrointestinal infection, chronic kidney disease, carbapenem resistance, age > 15 years, ischaemic heart disease, and hypertension (*P* < 0.0001, *P* = 0.0005, *P* = 0.0007, *P* = 0.0061, *P* = 0.0087, *P* = 0.0029, *P* = 0.0169, *P* = 0.0170 and *P* = 0.0462, respectively). The risk of mortality was 34% higher in adult patients and approximately 50% lower in paediatric patients (RR = 1.342; 95% CI: 1.063–1.669 vs. RR = 0.508; 95% CI: 0.275–0.889). Patients with multi-organ dysfunction were at a substantially high risk of death from *K. pneumoniae* BSI (RR = 16.67; 95% CI: 2.801–101.1 Septic shock and chronic kidney disease raised the RR three-fold, whereas ischaemic heart disease, mechanical ventilation, gastrointestinal infection, and carbapenem resistance raised it two-fold (Table 3).

**Table 3:** Outcomes among patients with *K. pneumoniae* bloodstream infection

Characteristics	Outcome					
	Deceased (n = 48)	Alive (n = 100)	RR	CI 95%		*P* value
*Age group*, n (%)						
Paediatric	10 (20.8%)	41 (41.0%)	0.5081	0.275 to 0.889		0.0169*
Adult	38 (79. 2%)	59 (59.0%)	1.342	1.063 to 1.669		0.0169*
*Category of multidrug resistance*, n (%)						
ESBL^1^ strains	16 (33.3%)	37 (37.0%)				0.7168
Carbapenem-Resistant strains	25 (52.1%)	26 (26.0%)	2.003	1.299 to 3.059		0.0029**
*Susceptible strains*, n (%)	7 (14.6%)	37 (37.0%)	0.394	0.188 to 0.778		0.0067**
*Invasive procedure*, n (%)						
CVC^2^	24 (50.0%)	49 (49.0%)				> 0.9999
MV^3^	29 (60.4%)	23 (23.0%)	2.627	1.719 to 4.024		< 0.0001****
Dialysis	5 (10.4%)	11 (11.0%)				> 0.9999
*Clinical presentation / complication*, n (%)						
Respiratory infection	21 (43.8%)	34 (34.0%)				0.2786
Gastrointestinal infection	18 (37.5%)	16 (16.0%)	2.344	1.315 to 4.142		0.0061**
Wound infection	2 (4.2%)	6 (6.0%)				> 0.9999
Urinary tract infection	7 (14.6%)	18 (18.0%)				0.8151
Septicaemia	12 (24. 5%)	38 (38.0%)				0.1392
Septic shock	17 (35.4%)	11 (11.0%)	3.220	1.655 to 6.262		0.0007***
Multi-organ dysfunction	8 (16.7%)	1 (1.0%)	16.67	2.801 to 101.1		0.0005***
*Underlying disease*, n (%)						
Diabetes	21 (43.8%)	35 (35.0%)				0.3660
Hypertension	24 (50.0%)	32 (32.0%)	1.563	1.034 to 2.316		0.0462*
Malignancy	18 (37.5%)	35 (35.0%)				0.8550
CKD^4^	12 (25.0%)	8 (8.0%)	3.125	1.394 to 6.982		0.0087**
ARDS^5^	6 (12.51%)	9 (9.0%)				0.5648
IHD^6^	11 (22.9%)	8 (8.0%)	2.865	1.256 to 6.496		0.0170*

Data are presented as a number of patients (n) with the corresponding percentage in parentheses (%). * P < 0.05; The statistical significance was indicated by a (*) symbol and the number of * represents the strength of the significance difference. ^1^ESBL, Extended-spectrum β-lactamases; ^2^CVC, Central venous catheter; ^3^MV, Mechanical ventilation ; ^4^CKD, Chronic kidney disease; ^5^ARDS, Acute respiratory distress syndrome ; ^6^IHD, Ischemic heart disease; RR, Relative risk; CI, Confidence interval

### Comparison between ICU and Non-ICU patients with *K. pneumoniae* BSI

A univariate analysis was conducted to compare the clinical characteristics and risk factors of patients with *K. pneumoniae* bloodstream infection related to ICU admission. Table 4 demonstrates the strength of the associations between the risk factors and ICU admission among *K. pneumoniae* BSI patients. Almost 40% of the patients with *K. pneumoniae* bloodstream infection were admitted to the ICU. Intriguingly, malignancy was significantly associated with non-ICU patients (*P* < 0.0001). In addition, the susceptible *K. pneumoniae* strains were significantly isolated from non-ICU patients compared to the ICU patients (*P* = 0.0108). Carbapenem resistance, mechanical ventilation, respiratory infection, multi-organ dysfunction, mortality, ischemic heart disease, and septic shock were significantly higher among ICU patients than non-ICU patients (*P* < 0.0001, *P* < 0.0001, *P* = 0.0006, *P* = 0.0039, *P* = 0.0043, *P* = 0.0133, and *P* = 0.0244, respectively).

**Table 4:** Comparison between ICU and Non-ICU patients with *K. pneumoniae* bloodstream infection

Characteristics	ICU Patients (n = 63)		Non-ICU Patients (n = 89)		*P* value
*Age group*, n (%)					
Paediatric	24 (38.1%)	27 (30.3%)			0.3838
Adult	39 (61.9%)	62 (69.6%)			0.3838
*Mortality*, n (%)	28 (45.9%)	20 (22.9%)			0.0043**
*Category of multidrug resistance*, n (%)					
ESBL^1^	17 (26.9%)	36 (40.5%)			0.1196
Carbapenem-Resistant	35 (55.6%)	20 (22.5%)			< 0.0001****
*Susceptible strains*, n (%)	11 (17.5%)	33 (37.1%)			0.0108*
*Invasive procedure*, n (%)					
CVC^2^	31 (49.2%)	44 (49.4%)			> 0.9999
MV^3^	38 (60.3%)	14 (15.7%)			< 0.0001****
Dialysis	7 (11.1%)	9 (10.1%)			> 0.9999
*Clinical presentation/ complication*, n (%)					
Respiratory infection	33 (52.4%)	22 (24.7%)			0.0006***
Gastrointestinal infection	10 (15.9%)	26 (29.2%)			0.0806
Wound infection	4 (6.4%)	5 (5.6%)			> 0.9999
Urinary tract infection	12 (19.1%)	14 (15.9%)			0.6652
Septicaemia	22 (34.9%)	29 (32.6%)			0.8618
Septic shock	18 (28.6%)	12 (13.5%)			0.0244*
Multi-organ dysfunction	8 (12.7%)	1 (1.1%)			0.0039**
*Underlying disease*, n (%)					
Diabetes	26 (41.3%)	36 (40.5%)			> 0.9999
Hypertension	27 (42.9%)	30 (33.7%)			0.3079
Malignancy	9 (14.3%)	46 (51.7%)			< 0.0001****
CKD^4^	9 (14.3%)	12 (13.5%)			> 0.9999
ARDS^5^	5 (5.6%)	10 (15.9%)			0.0521
IHD^6^	13 (20.6%)	6 (6.7%)			0.0133*

Data are presented as a number of patients (n) with the corresponding percentage in parentheses (%). * P < 0.05; The statistical significance was indicated by a (*) symbol and the number of * represents the strength of the significance difference. ^1^ESBL, Extended-spectrum β-lactamases; ^2^CVC, Central venous catheter; ^3^MV, Mechanical ventilation; ^4^CKD, Chronic kidney disease; ^5^ARDS, Acute respiratory distress syndrome; ^6^IHD, Ischemic heart disease

### Antimicrobial susceptibility of *K. pneumoniae* BSI isolates

**Fig. 1:**
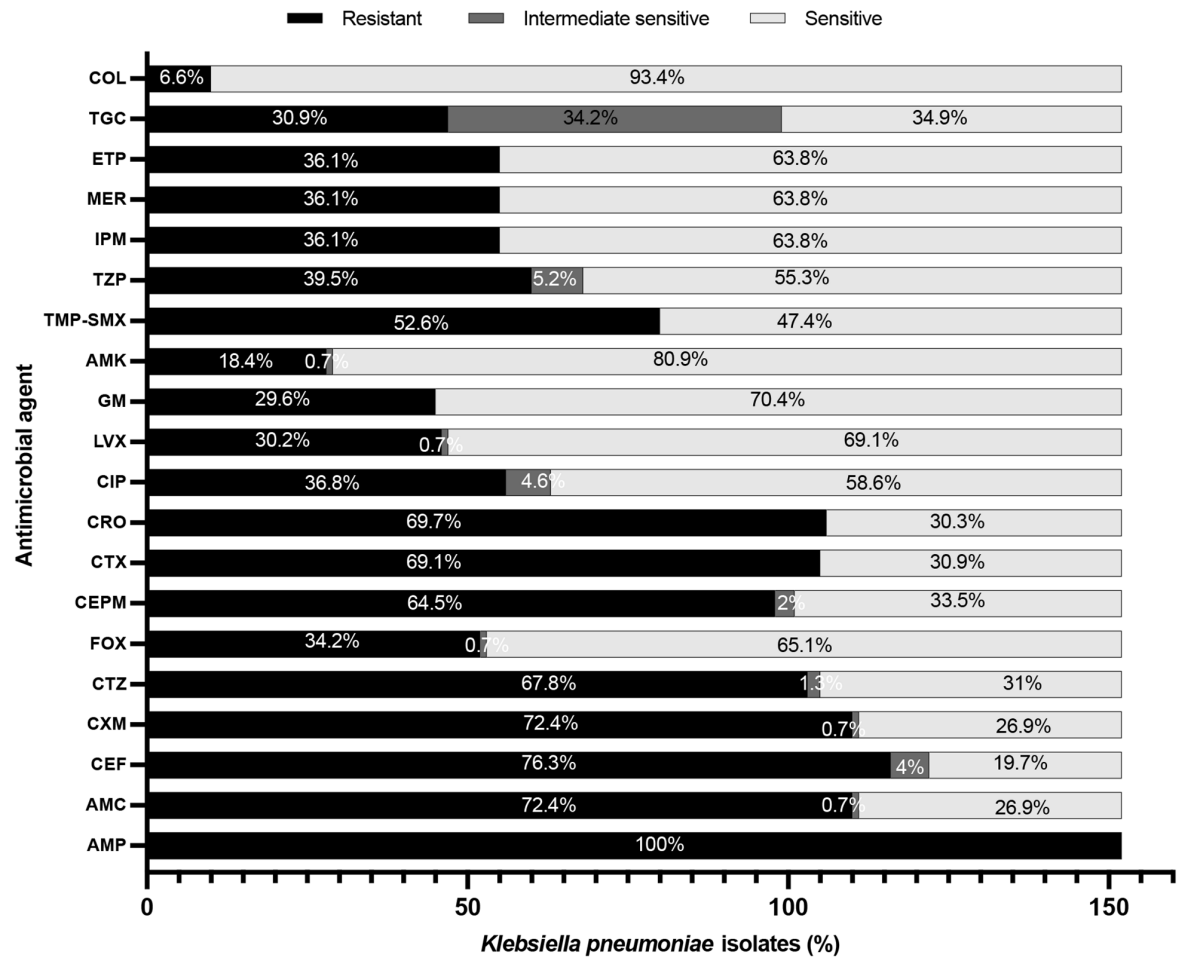
Antibiotic susceptibility of *Klebsiella pneumoniae* bloodstream isolates (n = 152). AMP, ampicillin; AMC, amoxicillin-clavulanate; AMK, amikacin; CEF, cephalothin; COL, colistin; CTZ, ceftazidime; CXM, cefuroxime; CFPM, cefepime; CTX, cefotaxime; CRO, ceftriaxone; CIP, ciprofloxacin; ETP, ertapenem; FOX, cefoxitin; GM, gentamicin; IPM, imipenem; LVX, levofloxacin; MER, meropenem; TGC, tigecycline; TMP-SMX, trimethoprim-sulfamethoxazole; TZP, piperacillin-tazobactam

Figure 1 summarises the results of the antimicrobial susceptibility tests. *Klebsiella pneumoniae* showed alarming resistance with XXXore than 60% of isolates were resistant to AMC as well as most of the cephalosporins (CEF, CXM, CTZ, CRO, CTX, and CEPM). Approximately half of the isolates were resistant to TMP-SMX. They exhibited lower resistance to the aminoglycosides (GM and AMK) than to the β-lactam antibiotics. Carbapenem-Resistant strains accounted for approximately 36% of all isolates. Interestingly, one-third of the isolates were resistant to tigecycline, a third were moderately sensitive, and a third were sensitive. Only isolates that were Carbapenem-Resistant were reported to have colistin resistance (6.6%), whereas isolates that were susceptible, ESBL, and Carbapenem-Resistant were reported to have tigecycline resistance.

## Discussion

*Klebsiella pneumoniae* BSIs are associated with high mortality rates worldwide. The emergence of antibiotic-resistant *K. pneumoniae* complicates the management of infections caused by these bacteria. This study aimed to evaluate the epidemiology, resistance profiles, and clinical outcomes of *K. pneumoniae* BSIs. The infection rate was slightly higher in females (52%) than in males (48%). The ICU and ER had the highest prevalence of *K. pneumoniae* BSI. ICUs are considered factories that create, amplify, and disseminate antibiotic resistance [20, 21]. The high prevalence of antibiotic resistance in ICUs might be due to multiple infections, frequent application of antimicrobials, or the frequent use of invasive procedures. In our study, *Klebsiella pneumoniae* BSIs in patients admitted to the ICU were significantly associated with multiple risk factors including carbapenem resistance, mechanical ventilation, respiratory infection, multi-organ dysfunction, ischemic heart disease, and septic shock were significantly higher among ICU patients than non-ICU patients. In a recent study by Wang et al. (2023), age over 70 years, admission to ICU, and urinary tract infection were found to be the risk factors for Carbapenem-resistant and ESBL-KP-resistance [22]. In another study by Huang et al. (2023), the risk factors for resistance to carbapenems in *K*. *pneumoniae* were ICU admission, respiratory failure, admission from the Emergency, and imipenem use [23].

In addition, the mortality rate was higher among ICU patients and contributed to 45.9% of the death rate. This finding is supportive of the EUROBACT-2 international cohort study on epidemiology and outcomes of hospital-acquired bloodstream infections in ICU patients which revealed predominant *Klebsiella* spp. (27.9%) bloodstream infection in ICU patients with poor outcomes [24].

Various hospital-based studies have suggested multiple comorbidities, including DM, biliary disease, and liver disease, as risk factors for *K. pneumoniae* BSI development [25, 26]. Here, we found that neurological disorders were the primary underlying conditions in both age groups, whereas DM was a main risk factor in adults. This conflicts with another study [28], which reported a lower risk associated with DM than with chronic liver disease and cancer. This contrast may reflect differences in the selected populations studied. Increasing age is associated with an increased risk of comorbidities [27, 28]. Here, the risk factors were associated with a 32.4% mortality rate. The risk of dying from *K. pneumoniae* BSIs was greater for adults than paediatric patients and was high for those with multi-organ failure. The higher mortality rate can be attributed to the greater virulence of the carbapenemase-producing strains, inappropriate antibiotic therapy, the greater toxicity and reduced effectiveness of antibiotics, and severe underlying diseases such as DM and chronic kidney disease [29].

Antimicrobial resistance is one of the most urgent public health concerns worldwide. Based on a comprehensive global analysis, it caused 1.27 million deaths in 2019, more than those caused by HIV/AIDS or malaria [30], and it could lead to 10 million deaths by 2050 unless a global effort to control it is implemented [31]. Increased prescription rates, and the extensive use of antibiotics, have led to the emergence of resistance against last-resort drugs, including carbapenems and colistin, especially among medically important bacteria such as *E. coli* and *K. pneumoniae*. It has been estimated that resistance to fluoroquinolones and β-lactam antibiotics, including carbapenems, cephalosporins, and penicillins, is responsible for more than 70% of deaths attributable to antimicrobial resistance [30]. The emergence and spread of MDR *K. pneumoniae* pose a global public health concern. In Saudi Arabia, the rate of *K. pneumoniae* resistance has increased substantially in the last few years, reaching 100% resistance in some regions [32]. We believe that the local pattern of antibiotic prescription is comparable to the national pattern of the Hafiz et al. (2023) study, which looks at the impact of improper antibiotic therapy on drug-resistant Gram-negative bacteria and indicates that it is strongly associated with poor outcomes [33]. Furthermore, according to worldwide research, inappropriate treatment is related to poor outcomes [34, 35].

ESBLs, produced primarily by gram-negative bacteria, mediate resistance to a wide range of β-lactam antibiotics, including extended-spectrum cephalosporins and the monobactam aztreonam. Most ESBL-encoding genes are carried by mobile genetic elements, facilitating the spread of resistance genes among bacteria. Several national and international studies have reported an increase in the prevalence of ESBL production among clinical isolates, reaching approximately 74% in some countries [36–38]. A study conducted in Saudi Arabia [39] revealed that, among the gram-negative bacteria, ESBL-producing *K. pneumoniae* was second only to *E. coli* as a cause of BSI. In the current study, 34.87% of the *K. pneumoniae* isolates produced ESBL, whereas this rate was 21.3% for the Makkah region (western Saudi Arabia) [32]. Similarly, the rate of ESBL-producing isolates was higher for Riyadh (central Saudi Arabia) than for Abha (an eastern region) and Al-Khobar (a central region) [40]. This regional variation in the prevalence of ESBL-producing *K. pneumoniae* in Saudi Arabia is presumably related to factors such as antibiotic prescription rates and resistance-related reporting. The rapid development of resistance is associated with membrane permeability, efflux pump activity, and β-lactamase production [41].

Our findings revealed high resistance (64–76%) to cephalosporins. Overall, our observed cephalosporin resistance rates were lower than those previously reported for Saudi Arabia: resistance to third- and fourth-generation cephalosporins increased substantially from 2011 to 2021, reaching 84.9%, 85.1% and 85.8% for ceftazidime, cefotaxime, and cefepime, respectively [32].

In this study, 36.18% of the isolates were Carbapenem-Resistant strains. Resistance to carbapenems is a major public health problem, as they are the last line of drugs for treating severe bacterial infections caused by ESBL-producing gram-negative bacteria. In Saudi Arabia, *K. pneumoniae* is the most common of the carbapenem-resistant Enterobacteriaceae found in hospitalised patients [42]. Unfortunately, the prevalence of carbapenem-resistant *K. pneumoniae* has significantly increased in Saudi Arabia: *K. pneumoniae* resistance to imipenem increased from 6.6% to 2011 to 59.9% in 2021, suggesting an increase in the consumption of antibiotics [32].

In 2019, *K. pneumoniae* was the second most common pathogen after *E. coli*, responsible for the most deaths attributable to antimicrobial resistance, causing > 600,000 antimicrobial resistance-associated deaths globally. In our study, mortality rates were higher for patients infected with ESBL- and Carbapenem-Resistant strains *K. pneumoniae* than for those infected with susceptible *K. pneumoniae* strains. BSI caused by ESBL-producing *K. pneumoniae* isolates were associated with 33.3% mortality, whereas higher mortality (52.1%) was associated with BSIs caused by Carbapenem-Resistant *K. pneumoniae* (52.1%), consistent with prior national studies [43, 44]. Globally, carbapenem-resistant *K. pneumoniae* was responsible for 55,700 deaths in 2019, followed by carbapenem-resistant *Acinetobacter baumannii*. Higher mortality is associated with infections caused by carbapenem-resistant *K. pneumoniae* than with those caused by carbapenem-sensitive *K. pneumoniae* [29]. A case-controlled study of critically ill patients found that carbapenem-susceptible *K. pneumoniae* BSI was responsible for 41% of in-hospital mortality, compared to 79% in those infected with Carbapenem-Resistant *K. pneumoniae* [45].

Our sample size was small, comprising only data collected over one year at KFMC. Using a larger sample will increase the precision of our findings with regard to statistical analysis. Additionally, further analysis is essential to examine the effects of the COVID-19 pandemic on the resistance profile of *K. pneumoniae*.

## Conclusions

*K. pneumoniae* isolates with a significant incidence of resistance were found in the institution. These results help shed light on microbial resistance and its relation to patient clinical outcomes. The high mortality rate among ICU patients is significantly associated with several risk factors, highlighting the importance of further investigating each factor and revising policies that might decrease the probability of such an outcome. Additionally, the observed resistance to colistin and tigecycline is concerning, particularly considering that a similar recent local study found a high prevalence of tigecycline resistance in ESBL-producing *K. pneumoniae* [36]. This may support the possibility that the selection pressure imposed by excessive antibiotic usage played a role in the development of the observed resistance.

## Data Availability

Data is available in the KFMC institute data system and could be available for the public upon special request through the correspondence author.
